# Recent Synthesis and Discovery of Brefeldin A Analogs

**DOI:** 10.3390/md16040133

**Published:** 2018-04-18

**Authors:** Seung-Mann Paek

**Affiliations:** College of Pharmacy and Research Institute of Pharmaceutical Sciences, Gyeongsang National University, Jinju daero 501, Jinju, Gyeongnam 52828, Korea; million@gnu.ac.kr; Tel.: +82-55-772-2424

**Keywords:** brefeldin A, medicinal chemistry, apoptosis, anticancer agent

## Abstract

The recent development of analogs of brefeldin A (BFA), a fungal metabolite, for the improvement of BFA apoptosis-inducing activity is described. BFA has been isolated from various soil or, more recently, marine fungi and has shown versatile beneficial activities. More importantly, the apoptosis-inducing activity of BFA in cancer cells highlights the possibility of further developing this natural product as an anticancer agent. Besides its biological importance, its structural features have also gathered tremendous interest from both medicinal and synthetic chemists. By a medicinal chemistry and total synthesis approach, numerous analogs from BFA have been developed to improve its inferior bioavailability and its antiproliferative ability. In this review, the recent medicinal chemistry efforts in relation to the production of BFA analogs are extensively presented.

## 1. Introduction

Since its first isolation from *Penicillium decumbens* in 1958 [1], brefeldin A (BFA) has been touted as a promising lead molecule in the world of drug development because of its potent biological activity in the antitumor [2], antifungal [3], and antiviral [4] fields. More importantly, its apoptosis-inducing properties [5,6] in various cancer cell lines and its ability to disrupt the *cis*-Golgi apparatus [7,8] drove medicinal chemists to discover more advanced pharmaceutical compounds (APIs) derived from BFA [9]. This medicinal promise drove the recent isolation of BFA from marine fungi, such as *Penicillium* sp. PSU-F44 or *Penicillium* sp. DT-F29, along with its other various structural analogs which were expected to be promising hits for drug development [10,11]. Unfortunately, its poor physical and pharmacokinetic (PK) properties hampered further development [12,13,14]. In response to these hurdles, a tremendous synthetic and medicinal chemistry effort has been put forth to improve its physical and PK properties, along with its own biological activities [15,16].

**Figure 1:**
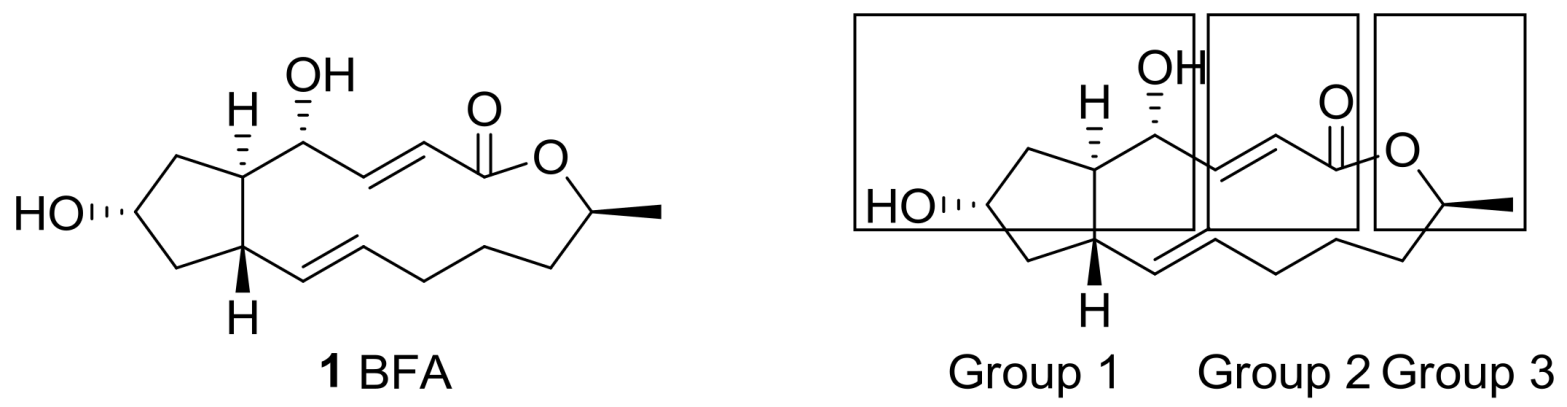
Structure of brefeldin A (BFA) and groups of its analogs based on structural modifications.

From a structural point of view, this natural product features a unique trisubstituted cyclopentane skeleton [17] with a 13-membered macrolactone ring that possesses two *trans*-alkene moieties. Because BFA also contains two hydroxyl groups, esterification was carried out at first for the purpose of producing prodrugs. (Group 1 in Figure 1) Differentiation of these two hydroxyl groups with similar steric demands was tried and showed a structure–activity relationship (SAR) on BFA. The electron-deficient alkene in the 13-membered lactone also provided a variety of synthetic opportunity for prodrug production. (Group 2 in Figure 1) Other approaches showed that structural changes on the terminal methyl group or skeletal changes on the BFA framework (Group 3 in Figure 1) could improve BFA activity efficiently. For this purpose, a systematic study from the simple esterification to the development of a synthetic route for BFA itself was carried out. Herewith, recent structural modifications of BFA and its SAR study will be presented.

## 2. Results

### 2.1. Modification of BFA

**Scheme 1:**
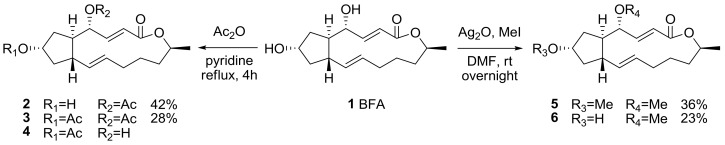
Modification of the two hydroxyl groups in BFA. DMF: dimethylformamide.

Early synthetic derivatization was executed of BFA itself to improve its physical properties. The production of a prodrug of BFA employing esterification or simple alkylation of two hydroxyl groups was usually pursued [14], although distinguishing between these two secondary hydroxyl groups was difficult in normal conditions, as shown in Scheme 1. For further SAR studies, however, this research by Hori group provided the basic information on the two hydroxyl groups [18]. Acetylation of BFA using acetic anhydride in refluxing pyridine afforded 4-acetyl BFA **2** and 4,7-diacetyl BFA **3** simultaneously. The compound 7-acetyl BFA **4** could be isolated after the treatment of BFA in refluxing acetic acid [19]. A similar modification was also observed in an *O*-methylated BFA synthesis. The compounds 4-*O*-methylated BFA **5** and 4,7-di-*O*-methylated BFA **6** could be obtained together and purified after treatment of BFA with silver oxide and iodomethane.

**Scheme 2:**
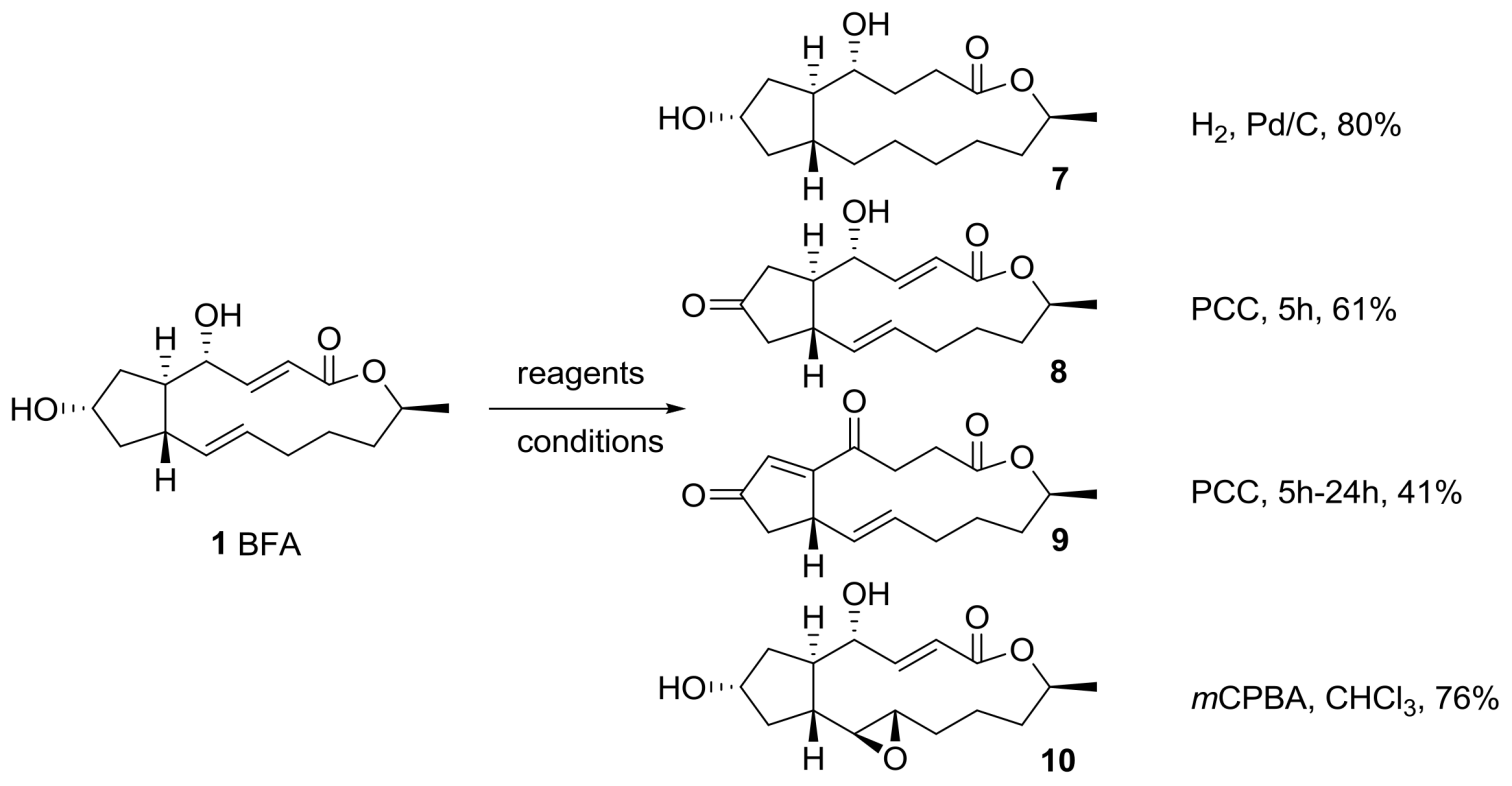
Oxidation and reduction of BFA. PCC: pyridinium chlorochromate; *m*CPBA: *meta*-chloroperoxybenzoic acid.

**Scheme 3:**
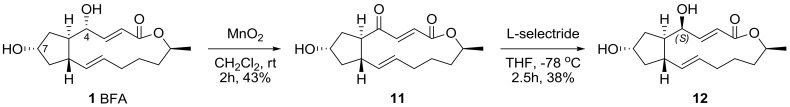
Chiral inversion of the 4-hydroxyl group in BFA. l-selectride: lithium tri-*sec*-butyl(hydrido)borate; THF: tetrahydrofuran.

Hori group also reported an oxidation–reduction protocol to afford BFA analogs in different oxidation states [20], as shown in Scheme 2. Conventional hydrogenation of BFA using H_2_, Pd/C produced conformationally free 13-membered lactone **7** in good yield. Pyridinium chlorochromate (PCC) oxidation of BFA also gave 7-keto BFA **8** in short time, while this condition gave isomerized 4,7-diketo BFA **9** over a prolonged reaction time. To add conformational rigidity, oxirane functionality was introduced into an electron-rich alkene in BFA, employing *meta*-chloroperoxybenzoic acid (*m*CPBA). This reaction proceeded in good yield and in a stereoselective manner:

An oxidation–reduction protocol was also utilized to inverse the chirality of the 4-hydroxyl group in BFA. The selective oxidation of allylic alcohol followed by a chemoselective reduction of BFA was performed, as shown in Scheme 3. Although each reaction condition produced BFA itself with no reaction from MnO_2_ oxidation (41%) or undesired reduction from l-selectride (52%), this reaction sequence produced 4*S*-BFA **12**, which is important for a systematic, original SAR study.

**Scheme 4:**
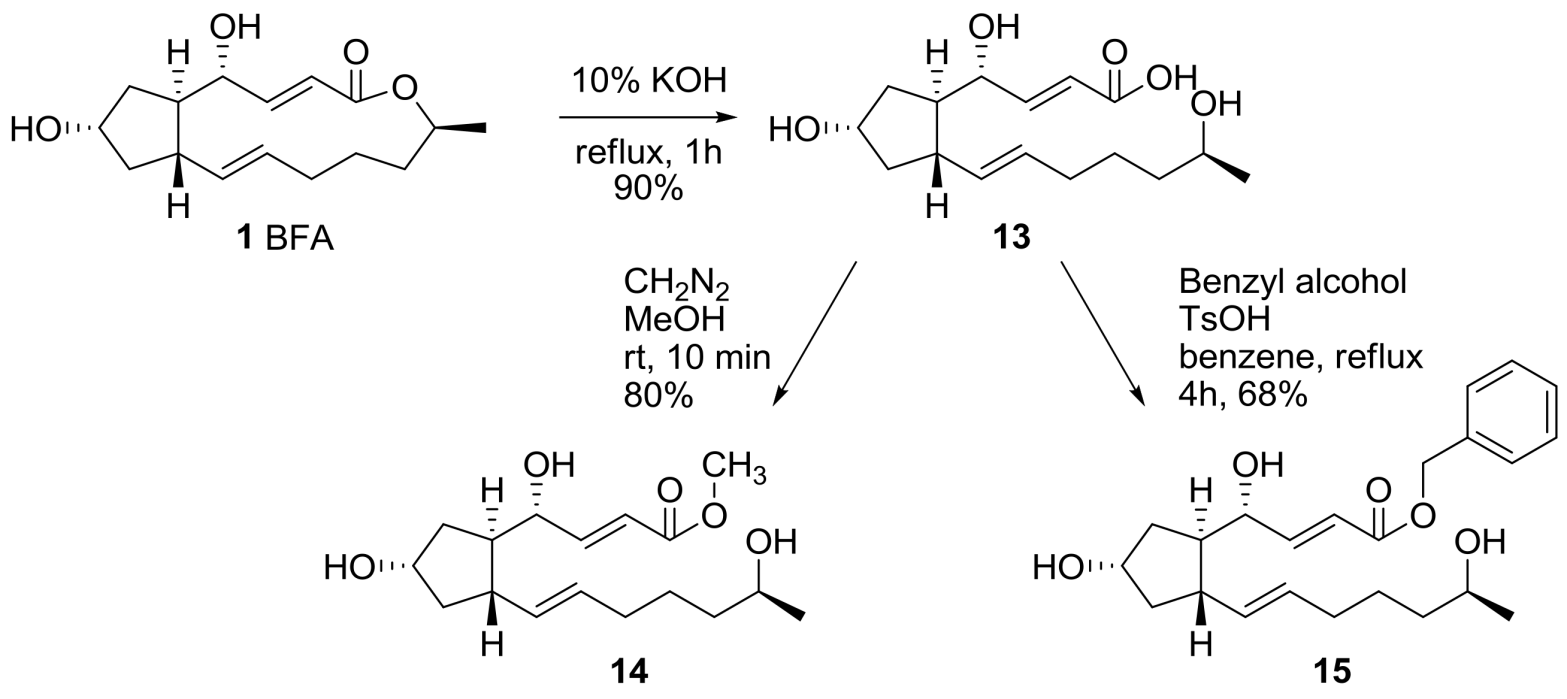
Modification of the carbonyl group in BFA.

The 13-membered lactone skeleton was hydrolyzed to find the relationship between ring conformation and activity in BFA (Scheme 4). BFA was hydrolyzed in basic conditions to produce seco acid **13** in good yield. The seco acid **13** was esterified again under *O*-methylation or in the Fischer esterification condition to make methyl ester **14** or benzyl ester **15**, which were tested for their biological activity.

The above synthesized analogs were tested for their apoptosis-inducing activity using HCT-116 cells, as shown in Table 1. After treatment with the analogs, characteristic DNA ladder formation was observed, as found also following treatment with BFA. The minimum effective concentrations (EC) of the analogs showed that 7-acetyl BFA **4** was almost equipotent to BFA, while 4(*S*)-BFA **12** lost BFA activity. The cytotoxicity of the analogs and their IC_50_ were also evaluated. On the basis of the similar cytotoxicity of mono-acetyl BFA derivatives such as **2** or **4** compared to BFA, it can be envisioned that a prodrug approach of the hydroxyl group in BFA would be a promising strategy to find advanced cytotoxic agents. It also seems that the active conformation needs two alkene groups and a 13-membered lactone framework, since the saturated BFA **7**, epoxy BFA **10**, ring-opened BFA **13**, **14**, and **15** showed lower apoptotic or cytotoxic activities compared to BFA.

**Scheme 5:**
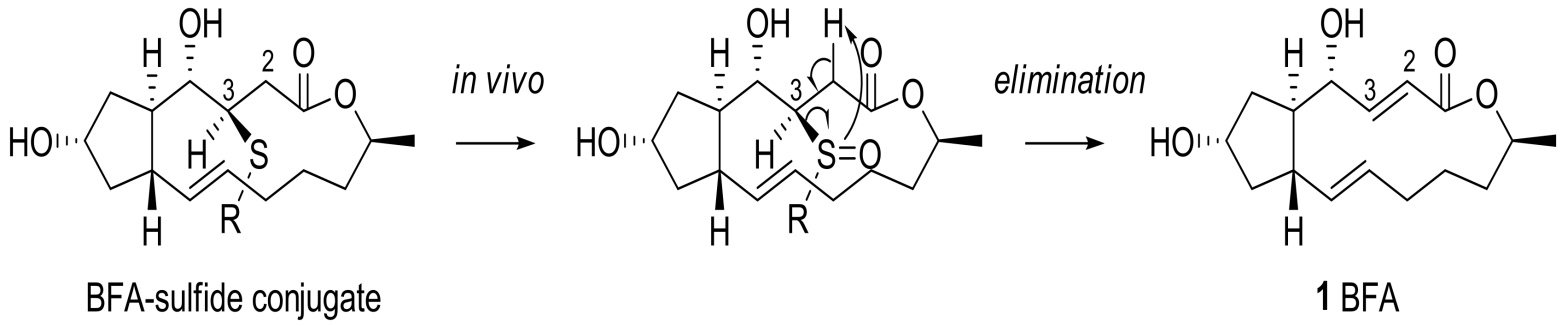
Sulfide conjugate approach for BFA.

Another medicinal chemistry approach was employed to produce a water-soluble prodrug. For this purpose, Cushman group tried to change the C2–C3 alkene functional group of BFA with various sulfide moieties to produce a latent leaving group via in vivo oxidation and *syn*-elimination [21], as shown in Scheme 5. The addition of various thiols was tried in order to improve BFA activity and water solubility, to proceed with a study of facial selectivity.

**Scheme 6:**
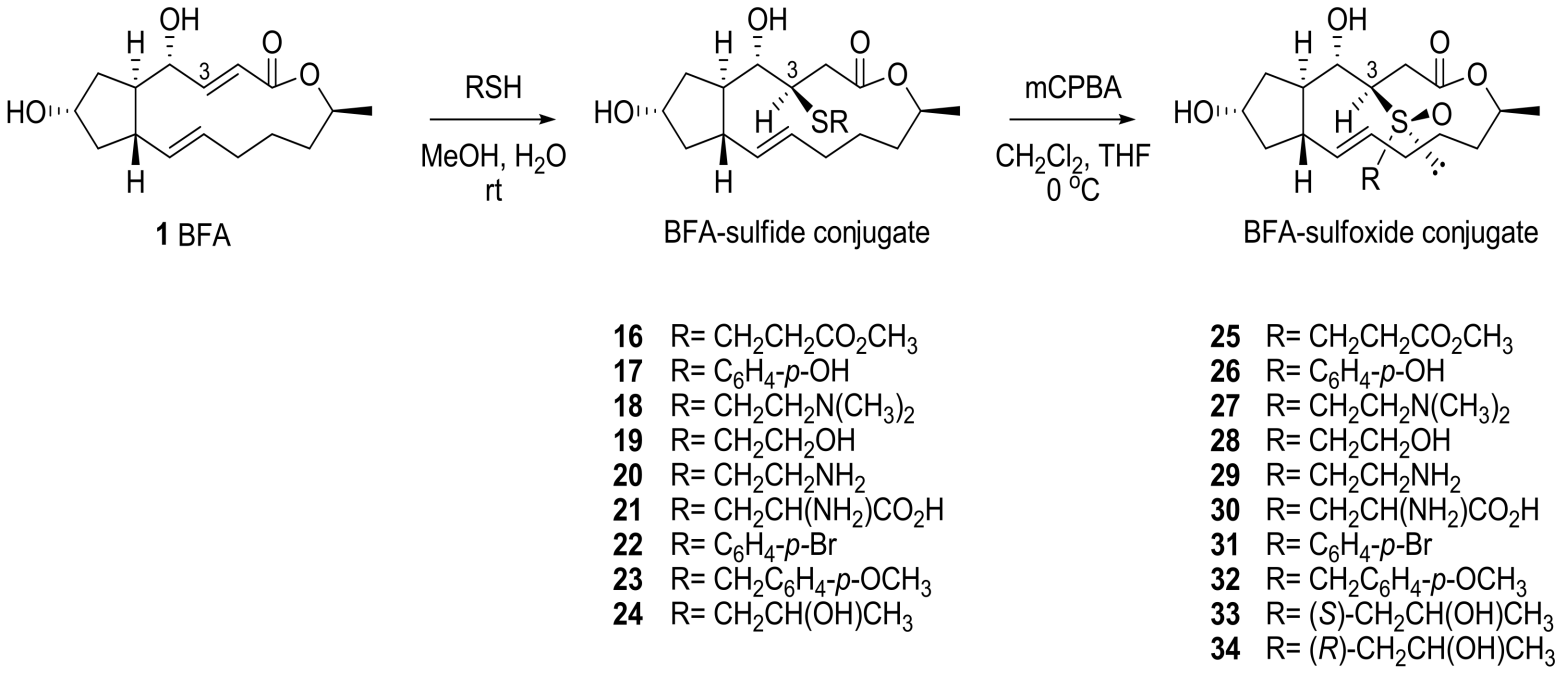
Synthesis of sulfide- and sulfoxide-conjugated BFA analogs.

BFA was treated with various thiols under basic conditions to provide conjugated sulfide adducts which were oxidized to sulfoxides in the presence of alkene (Scheme 6). Although excess amounts or a prolonged reaction time created a correspondingly overoxidized product, such as sulfone or epoxide, careful treatment of the reaction usually produced the desired sulfoxide in moderate yield. The newly generated stereocenters at C3 and sulfur were determined to be (*R*) and (*S*), respectively, on the basis of X-ray crystallography (Nonius Kappa CCD diffractometer) of **22** and **32** [22]. Because the addition process occurs very selectively, a diastereomeric mixture of these additional products could be easily purified by silica gel chromatography. (*S*)- or (*R*)-configured side chain preparations of the adducts **33** or **34** were obtained by the corresponding chiral thiol addition.

For in vitro assays, these analogs were used to treat various cancer cell lines from the National Cancer Institute repository [23]. As shown in Table 2, the sulfide analogs were less potent than the corresponding sulfoxide analogs. This tendency means that the sulfoxide analogs would easily be converted to BFA in the cells, while the sulfide analogs would need additional oxidation to provide BFA. When the elimination rate was analyzed in a crude NMR study, **33** (*t*_1/2_ 23 min) and **34** (*t*_1/2_ 26 min) were more rapidly converted to BFA than **31** (*t*_1/2_ 440 min). Although the sulfide analogs did not show potent anticancer activity in in vitro assays, the results suggested the possibility of another prodruggable strategy because the sulfide moiety can be easily oxidized with CYP450 or Flavin monooxygenase in vivo. It is important to note that the water solubility of these analogs improved in relation to BFA. For example, cysteine-conjugated analog **21** was more soluble (35 mg/mL) than BFA itself (2.8 mg/mL).

**Scheme 7:**
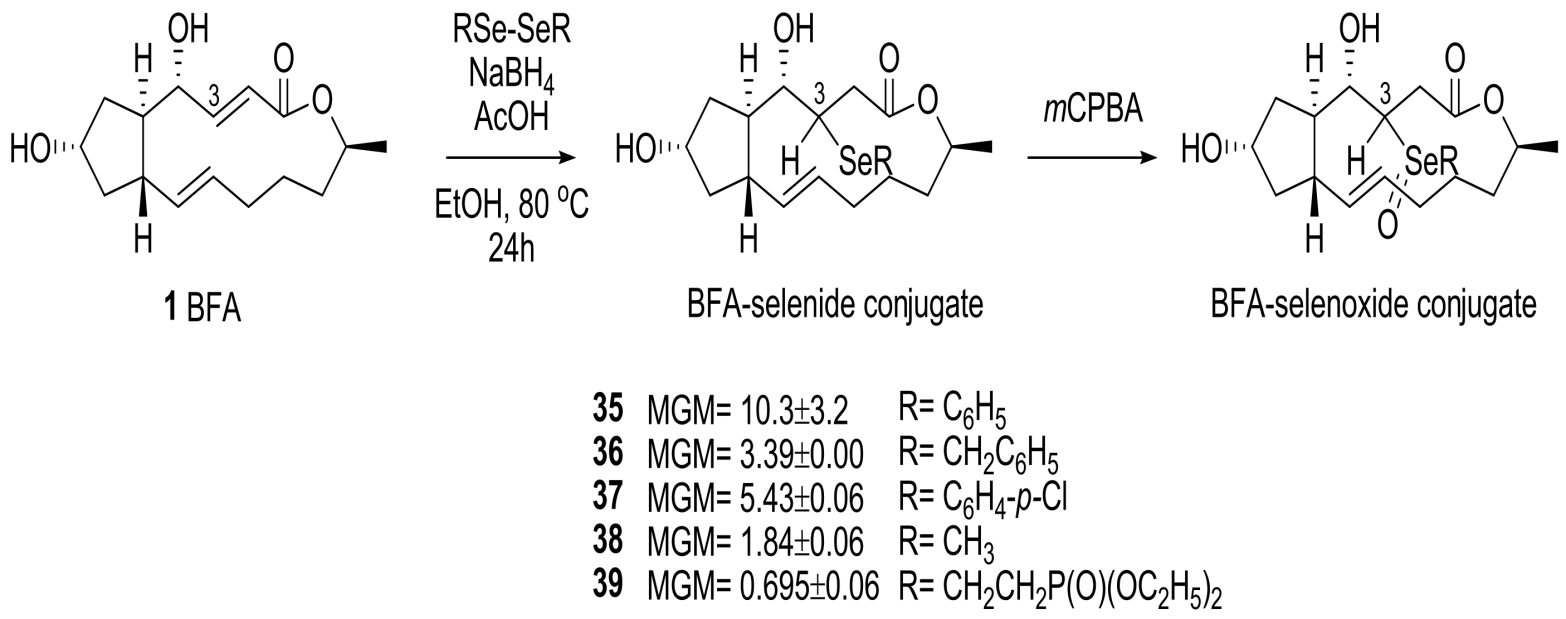
Synthesis of selenide-conjugated BFA analogs and their MGMs.

For more promising analogs, selenide-conjugated analogs were also prepared using a similar strategy. In Scheme 7, *m*CPBA oxidation of the selenides **35** produced the corresponding selenoxides (61%) along with BFA (38%), and this emphasized the higher lability of the selenide functional group for *syn*-elimination. This reactivity could be utilized for the production of another potential BFA prodrug.

**Scheme 8:**
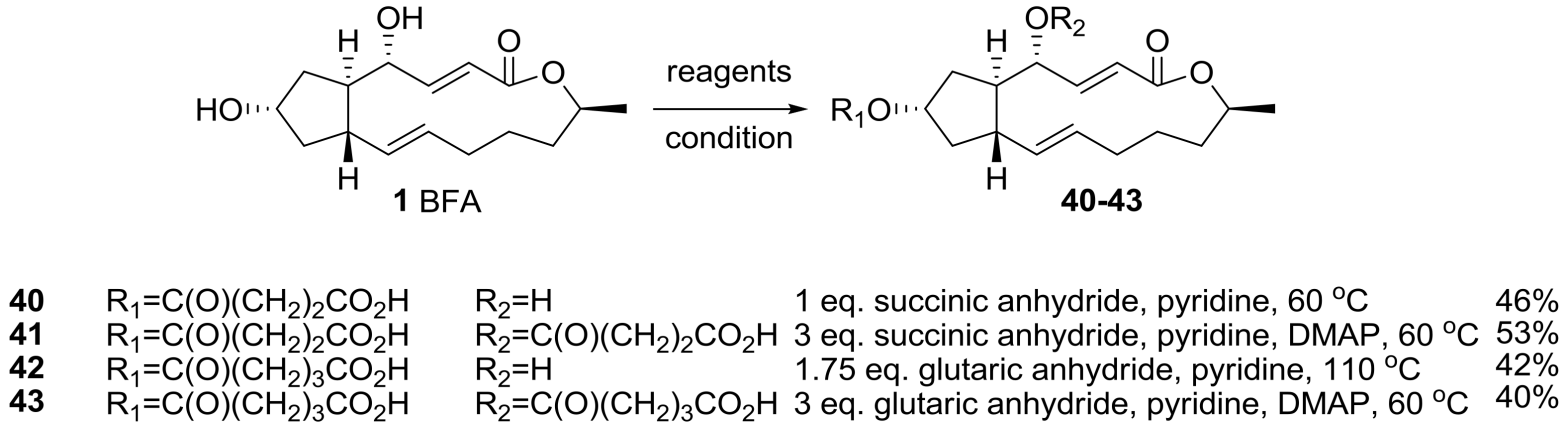
Synthesis of succinyl or glutaryl BFA analogs.

Cushman group also reported an ester analog of BFA [24], as shown in Scheme 8. It was envisioned that a long lipid chain may present the possibility to use an affinity column preparation to directly find a binding site. Because the biological response of BFA was unique and complex depending on the cancer cell line, a target fishing strategy using an affinity column was desired to find BFA’s possible biological mechanisms of action. However, the presence of two secondary hydroxyl groups made it difficult to synthesize a specific and active affinity matrix. In addition, the modification of the two hydroxyl groups could affect the SAR study. So, various alkyl chains should be linked to BFA not only to find target sites, but also to discover active analogs. A succinyl or a glutaryl group was attached to BFA via reaction condition screening.

**Scheme 9:**
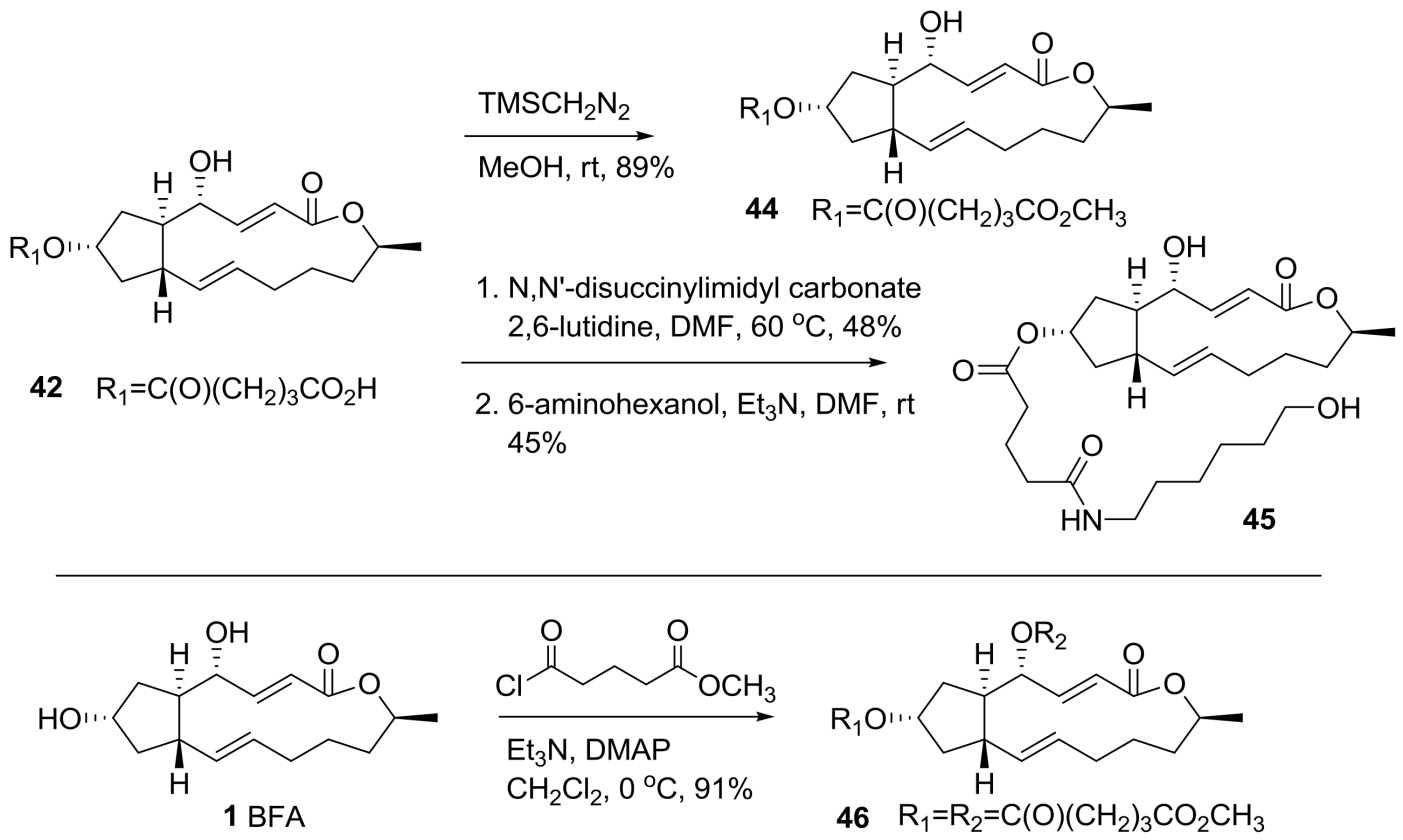
Synthesis of BFA analogs with long lipid chains. TMS: trimethylsilyl; DMAP: 4-dimethylaminopyridine.

Further modifications for SAR studies and affinity matrix preparation were executed as shown in Scheme 9. A simple esterification with TMSCH_2_N_2_ was carried out to give ester analog **44** in good yield. More importantly, a longer lipid chain was introduced by the activation of the carboxylic acid in **42** and amide coupling with 6-aminohexanol, to provide hydroxyl analog **45**. This analog could be utilized for affinity matrix preparation using biotin or resin with the purpose of finding a target site in cells so long as this analog does not lose BFA own biological effect on cancer cell lines. Bisfunctionalization of 4,7-dihydroxyl BFA was also carried out via a simple esterification reaction.

**Scheme 10:**
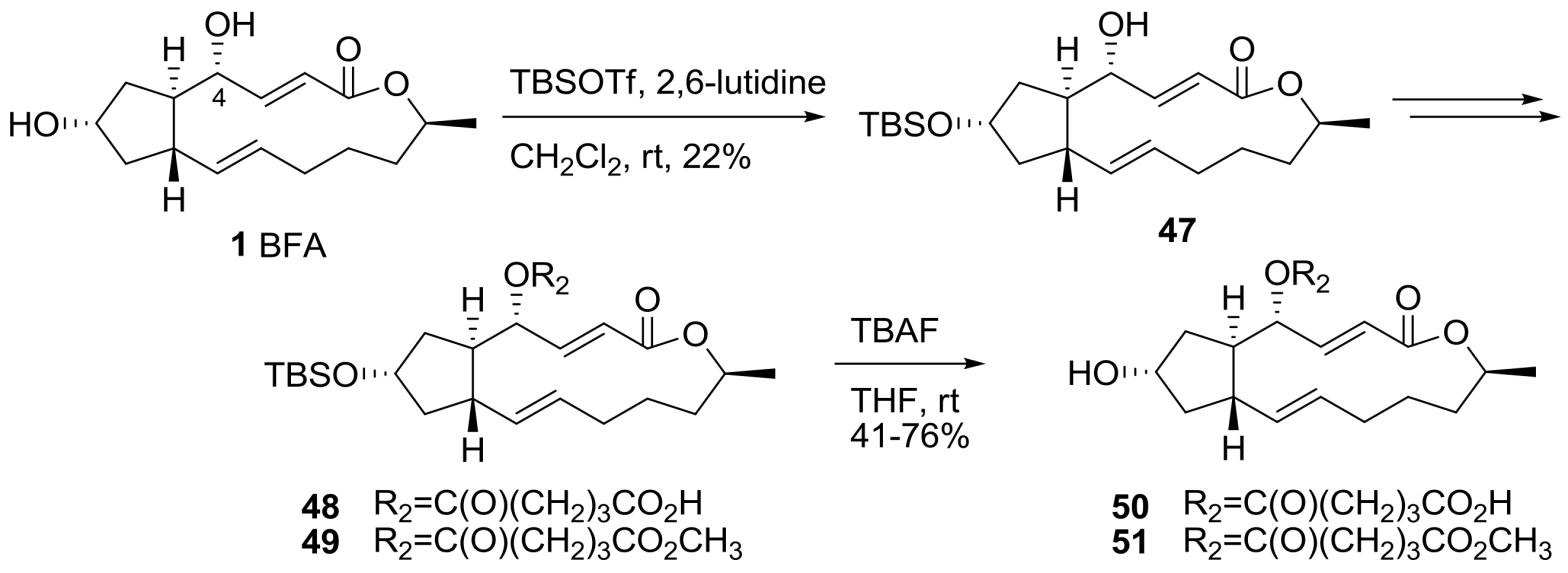
Synthesis of 4-acyl BFA analogs. TBS: *t*-butyldimethylsilyl, Tf: trifluoromethanesulfonyl, TBAF: tetra-*n*-butylammonium fluoride.

Scheme 10 describes the monofunctionalization at the 4-hydroxyl group in BFA. A protection reaction was necessary for selective modification, because the 7-hydroxyl group was more reactive than the 4-hydroxyl group. *t*-butyldimethylsilyl (TBS) protection afforded monosubstituted BFA **47** in low yield. With this 7-protected BFA prepared, similar functionalizations were executed to give the desired monosubstituted analogs at the 4-hydroxyl group in BFA after conventional TBS deprotection.

The biological evaluation of the synthesized ester analogs is depicted in Table 3. The mean graph midpoint (MGM) of the GI_50_ value for various cancer cell lines was calculated and reported. Although a solid SAR could be established from this result, the cell permeability of the analogs is important for their anticancer activity, as demonstrated by the fact that methyl ester analogs were usually superior than their carboxylic acid analogs. It is impressive that the anticancer activity was still maintained in the long chain-extended analog **45**. This analog could be utilized in affinity matrix preparation.

**Scheme 11:**
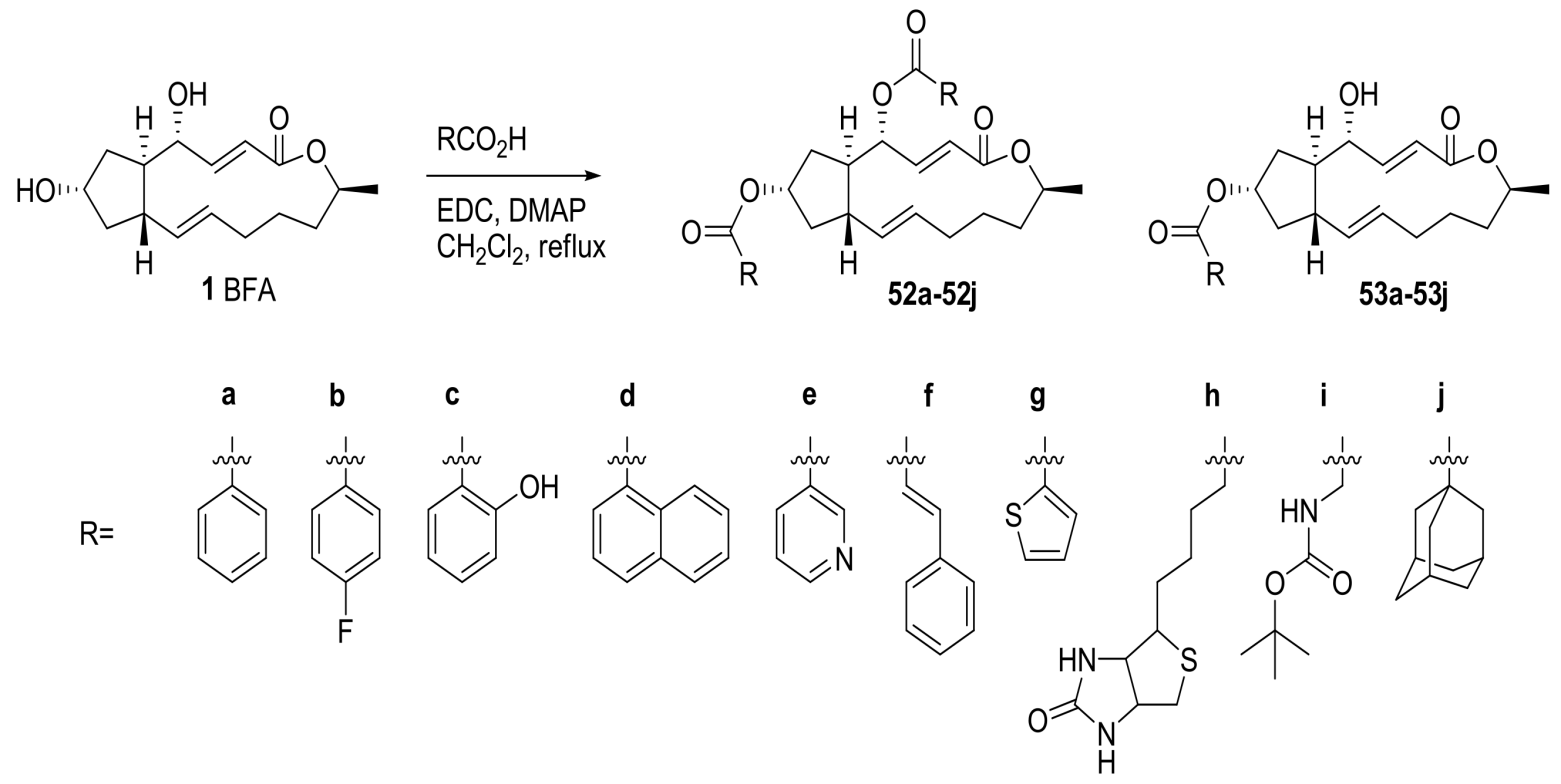
Synthesis of BFA analogs with ester functional groups. EDC: 1-Ethyl-3-(3-dimethylaminopropyl)carbodiimide.

More various functional groups, including aromatic esters, were also attached to BFA to evaluate their anticancer activity. Zhu group reported the synthesis of a BFA-based prodrug via simple esterification with various aromatic or alkyl groups, as shown in Scheme 11 [9]. These analogs were evaluated in TE-1 cell line, showing moderate activity for the analogs **52a**, **53a**, and **53h**. Because the in vivo stability of BFA hampered its further development, a stability test of the analog **53h** was also carried out to find if it could be utilized for BFA prodrug production. 

**Scheme 12:**
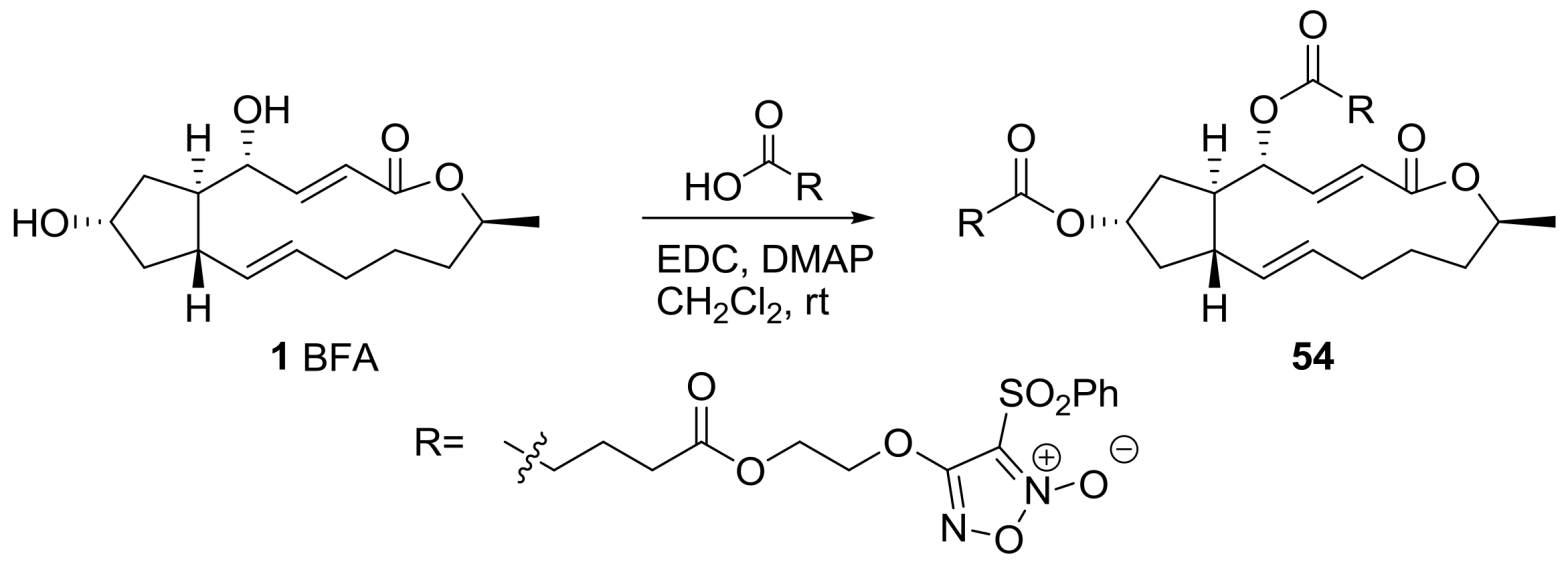
Synthesis of NO-producing ester analogs of BFA.

A recent impressive BFA analog was developed using a nitric oxide (NO)-producing moiety [25], as shown in Scheme 12. Since an NO donor hybrid was used for chemotherapy, it has been employed to strengthen the cytotoxicity level of anticancer agents such as evodiamine or spirolactone-type diterpenoid. Furoxan, a NO-producing subunit, was attached to BFA on the basis of SAR. After extensive screening and optimization of conditions, the compound **54** was found to be active against prostate, colon, and liver cancer cell lines with IC_50_ of nanomolar concentration. This cytotoxicity is consistent with the high NO-producing ability of **54** in HepG2 (liver cancer) cells. It is also important that **54** induced apoptosis of HepG2 cells in a dose-dependent manner, and apoptosis was reduced when hemoglobin, an NO scavenger, was present. This means that an NO-producing ability is important for the induction of apoptosis in cancer cell lines. The employment of a protein array indicated that survivin and heme oxygenase-1 and 2 are related to this apoptotic response of BFA analog **54**.

**Scheme 13:**
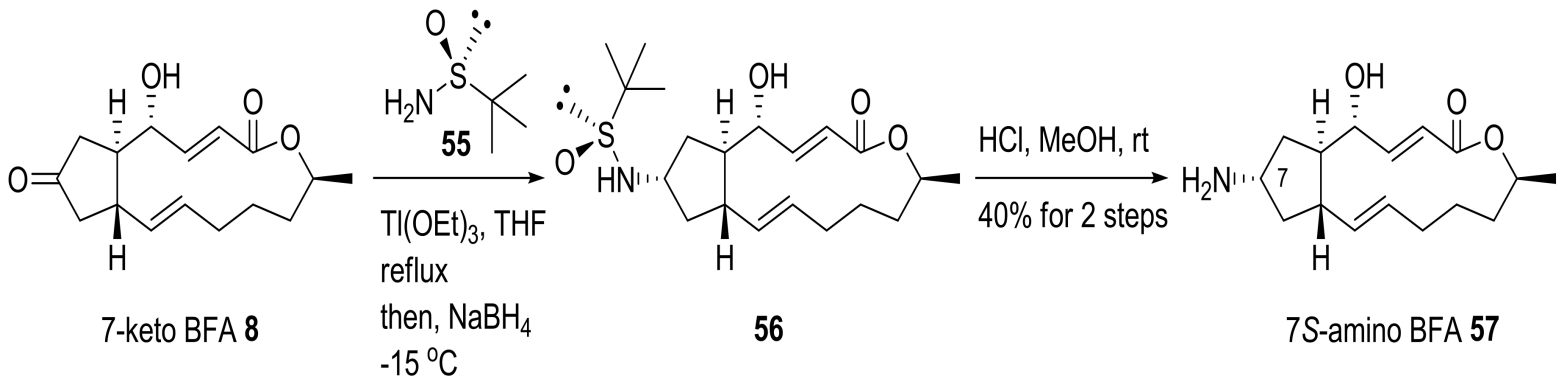
Synthesis of 7-amino-BFA analogs.

Amino-BFA analog **57** was prepared using reductive amination of 7-keto BFA **8** and chiral sulfinyl amine **55**, followed by acidic deprotection [26], as shown in Scheme 13. However, when **57** was used to treat HeLa cells to observe its disrupting effect on the Golgi complex, it showed less activity than BFA itself.

### 2.2. Modification of BFA Based on Total Synthesis

BFA gathered tremendous attention from synthetic chemists because of its unique structure. A fused bicyclic skeleton with trisubstituted cyclopentane and 13-membered lactone has been always a synthetic challenge for full synthetic approaches. In addition, the presence of two alkenes and four stereocenters was also a formidable hurdle in the synthesis of BFA. To solve these problems, many synthetic routes have been developed by employing unique methodologies or multiple-step synthesis, which also provided analogs of BFA. It is worth noting that synthetic analogs from total synthesis of BFA may possess a skeleton different from the BFA’s original one. Since conventional medicinal chemistry approaches used BFA as a starting material, simple modifications, such as esterification–hydrolysis, oxidation–reduction, and addition reactions of BFA were possible. The construction of the BFA skeleton via total synthesis from simple starting materials, however, could change the BFA’s skeleton itself. This dramatic change may lead to precious information for an SAR study.

**Scheme 14:**
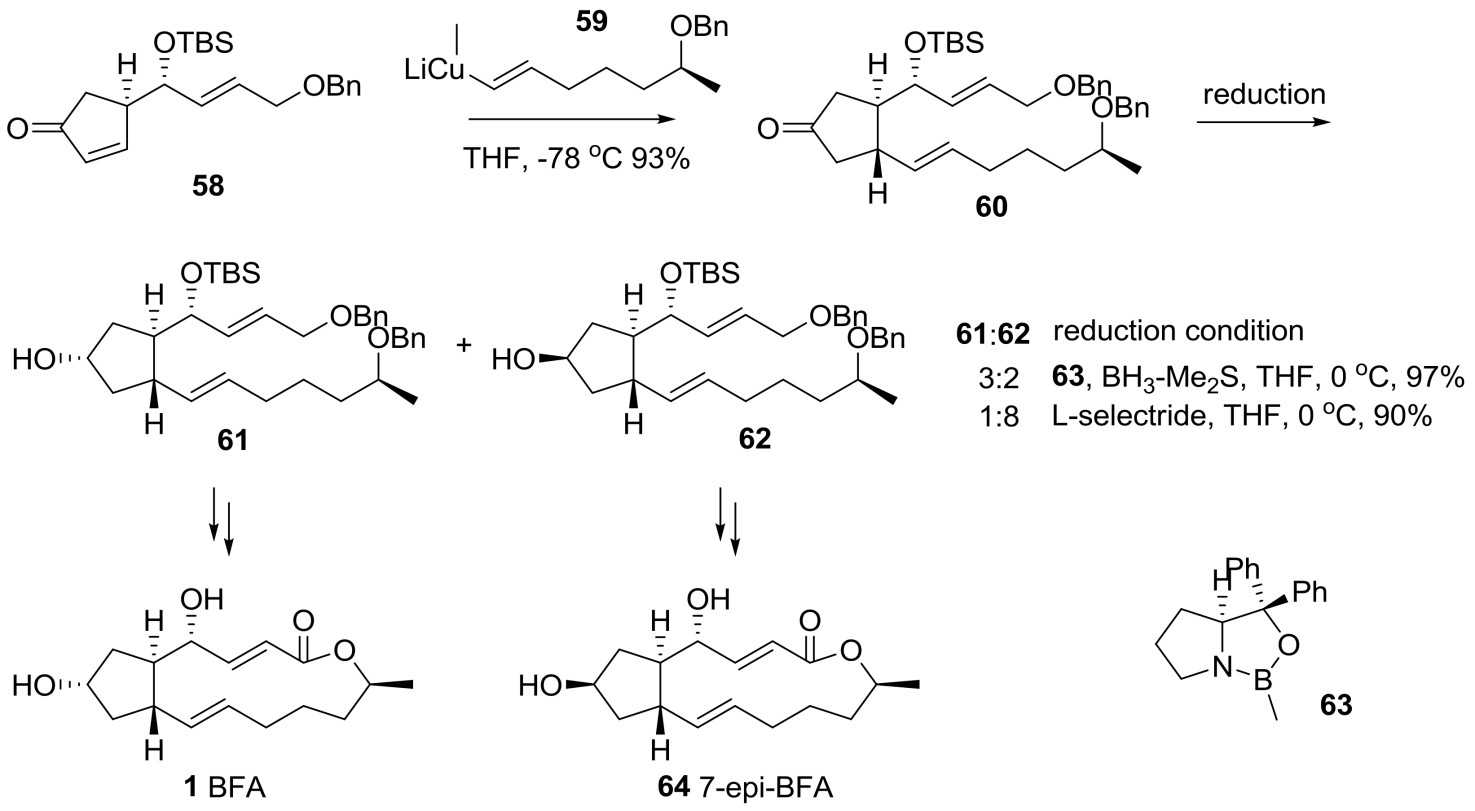
Total synthesis of BFA and 7-epi-BFA using the conjugated addition route.

Wu group reported a conjugate addition of vinyl cuprate **59** to substituted cyclopentenone **58** to build another side chain in the BFA framework [27], as shown in Scheme 14. While an achiral reduction afforded the undesired epimer **62** as a major product, a chiral reduction using (*S*)-CBS reagent **63** was carried out to produce the desired secondary alcohol **61** as a major product. The desired alcohol **61** and its epimer **62** were transformed into BFA **1** and 7-epi-BFA **64**, following the conventional reaction route.

**Scheme 15:**
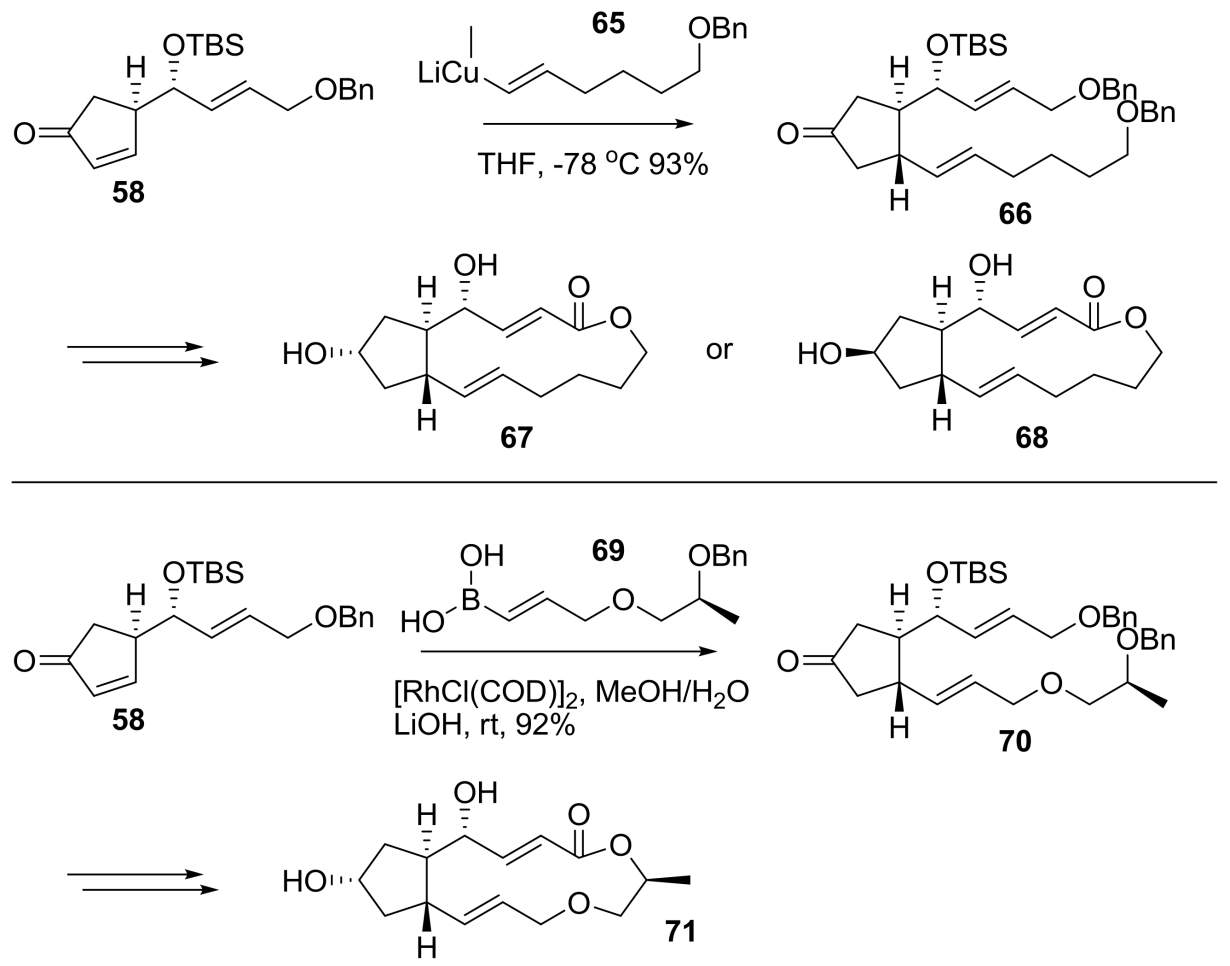
Synthesis of NO-producing ester analogs of BFA. COD: cyclooctadiene.

After the development of a synthetic route to BFA, its variation using different synthon was tried to make BFA analogs, as shown in Scheme 15 [28]. Desmethyl BFA **67** and its 7-epimer **68** were prepared from a similar reaction route, employing desmethyl side chain **65** instead of **59**. A similar strategy was also tried for 13-oxo-BFA analog **71** synthesis [29]. Using vinylborane **69** from lactic acid, an Rh-mediated conjugate addition was carried out to attach a side chain in the BFA skeleton. Following a similar reaction pathway, 13-oxo-BFA analog **71** could be obtained uneventfully. It is interesting that the same strategy was also applied to pursue 13-thio-BFA, which could not be synthesized via an Rh-mediated coupling reaction.

**Scheme 16:**
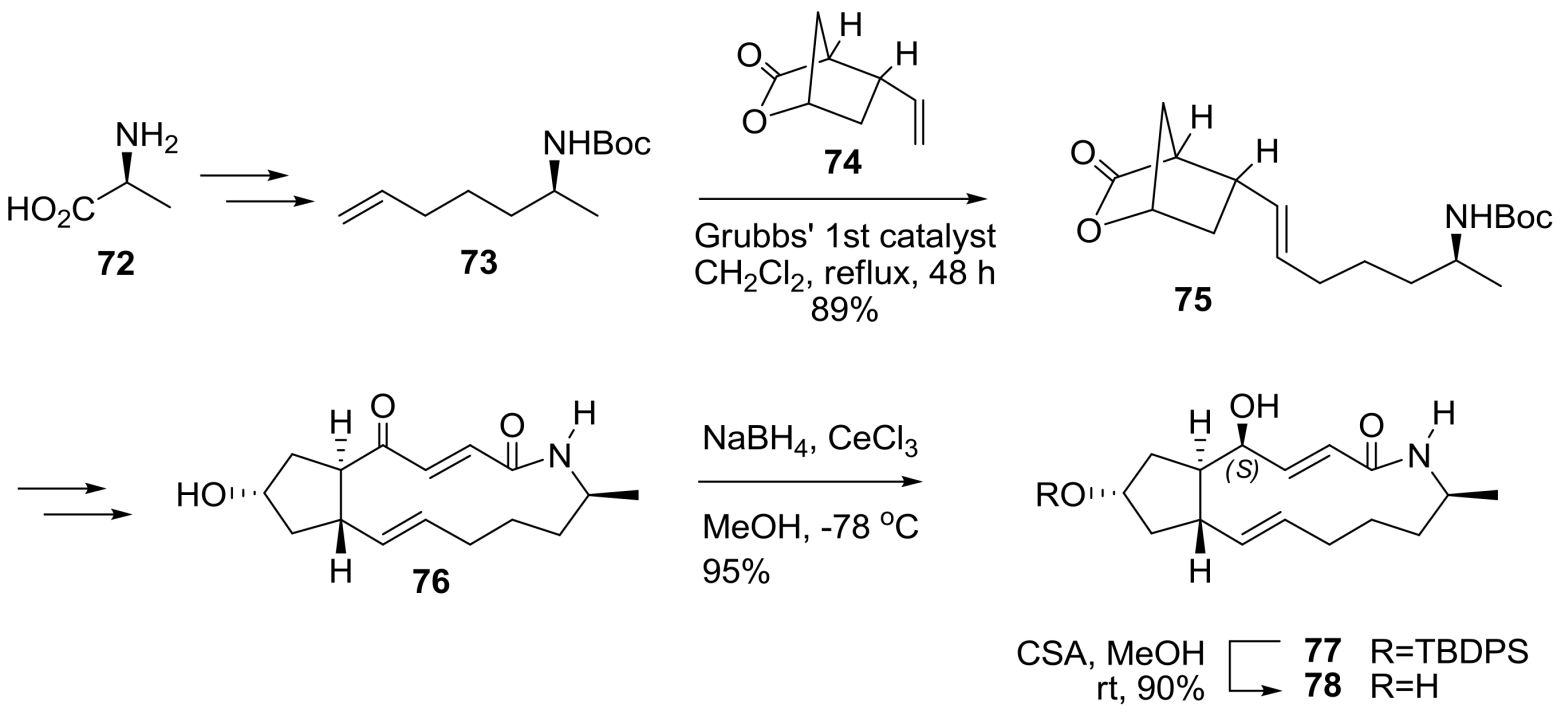
Synthesis of 4-epi-BFA lactam analogs. Boc: *t*-butoxycarbonyl, CSA: camphorsulfonic acid, TBDPS: *t*-butyldiphenylsilyl.

Amino-BFA was also prepared in Scheme 16. To introduce nitrogen in the BFA structure, alanine **72** was chosen as the starting material for side chain **73**. After efficient cross metathesis (CM) [30] with side chain **73** and bicyclic lactone **74**, the desired CM adduct **75** was successfully transformed to unsaturated enone **76**. For the completion of the synthesis, a Luche reduction was tried to establish a 4*R* chiral alcohol moiety. However, this reaction condition, surprisingly, afforded the undesired epimer **77** as a single diastereomer. After an extensive survey of the reaction conditions and structural confirmation, the synthetic program ended with the synthesis of the 4-epi-BFA lactam **78** analog [31]. 

**Scheme 17:**
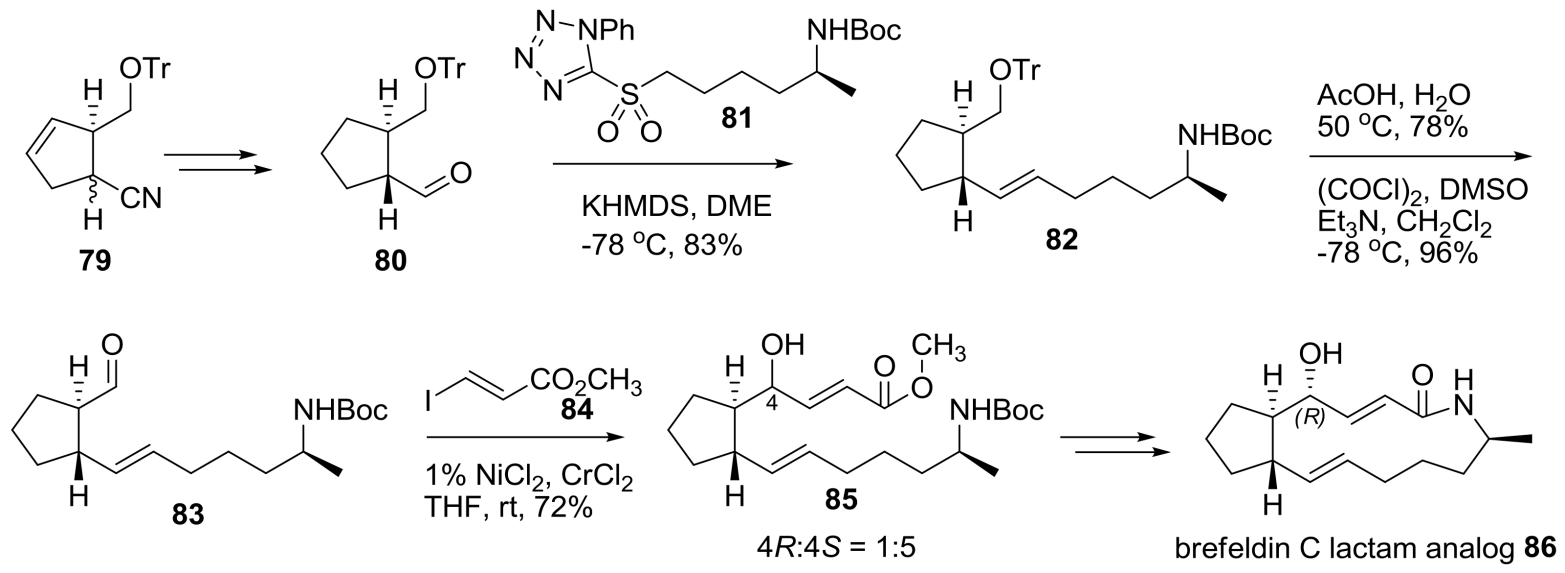
Synthesis of brefeldin C lactam analogs. Tr: triphenylmethyl, KHMDS; Potassium *bis*(trimethylsilyl)amide, DME: dimethoxyethane, DMSO: dimethyl sulfoxide.

Another approach to prepare a lactam analog of brefeldin was tried using a different synthetic route as shown in Scheme 17 [32]. Helmchen group developed an efficient synthesis of substituted cyclopentene **79** using catalytic allylic alkylation of cyanomalonate with chiral iridium as a catalyst. After hydrogenation, semi-reduction, and epimerization, chiral aldehyde **80** was obtained quickly and submitted to Julia-Kocienski olefination with sulfonyltetrazole **81** to produce *trans*-alkene **82** in good yield. Treatment of **82** with acidic deprotection and Swern oxidation afforded the resulting aldehyde **83**, which was then submitted to Nozaki-Hiyama-Kishi (NHK) alkenylation with iodoacrylate **84**, with nickel/chrome as a catalyst. Although this reaction gave the undesired *Si*-face product as a major isomer, with a small amount of the desired *Re*-face adduct, it could be easily and successfully converted to 7-deoxy brefeldin A (brefeldin C) lactam analog **86**.

**Scheme 18:**
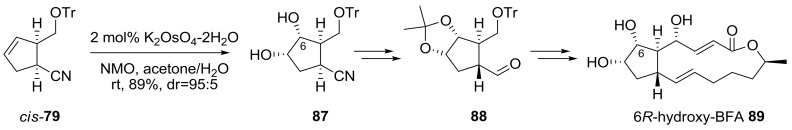
Synthesis of (*6R*)-hydroxy-BFA analogs. NMO: *N*-methylmorpholine *N*-oxide.

Instead of hydrogenation, dihydroxylation of cyclopentene **79** was carried out to introduce a hydroxyl group at the C6 position [26], as shown in Scheme 18. Two substituents of cis-**79** possessed a β-face of the cyclopentene plane, and directed catalysis of potassium osmate occurred on the α-face dominantly. With dihydroxyl cyclopentane **87** prepared, a similar conversion, as in Scheme 17, including semi-reduction, epimerization, Julia-Kocienski olefination, and Nozaki-Hiyama-Kishi alkenylation was executed to produce the (*6R*)-hydroxy BFA analog **89**. It is impressive that Golgi complex redistribution was observed in mammalian or plant cells when **89** was used to treat the cells, as observed for BFA [33].

**Scheme 19:**
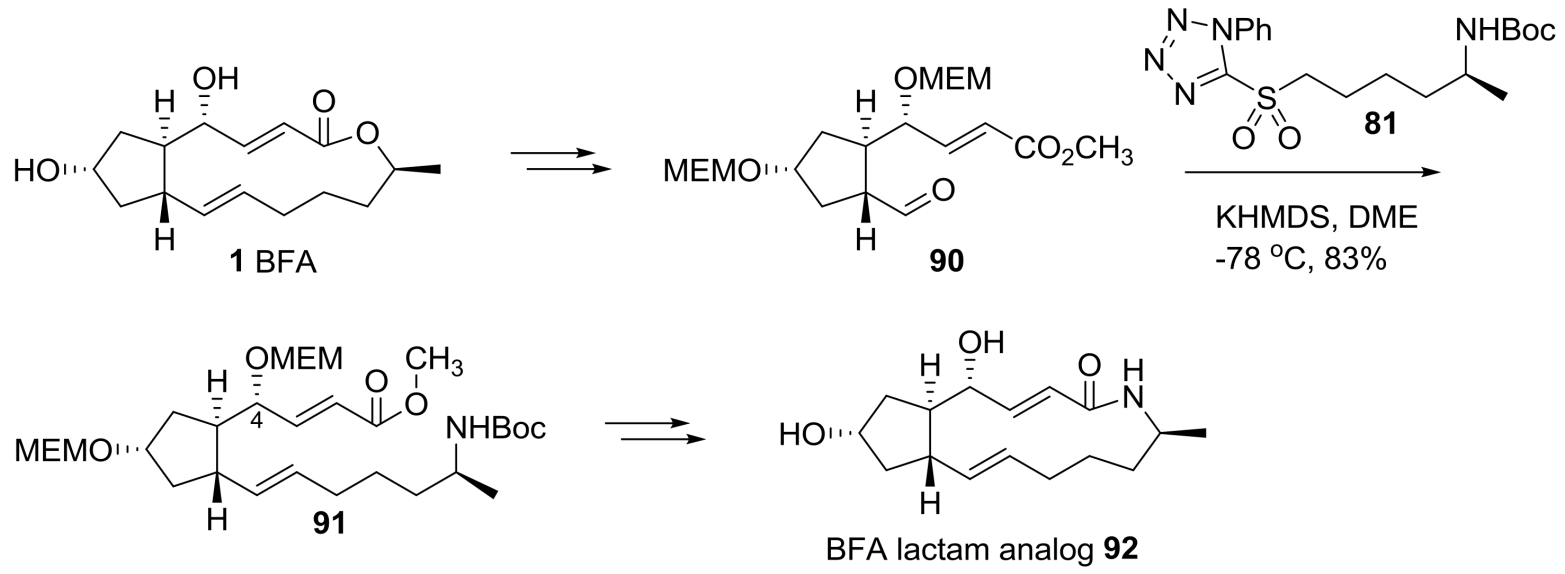
Synthesis of BFA lactam analogs. MEM: 2-methoxyethoxylmethyl.

Helmchen group also reported the semi-synthesis of BFA analogs using hybridization of a BFA skeleton and artificial side chains [26], as shown in Scheme 19. Starting from BFA, protection and chemoselective olefin cleavage of the electron-rich olefin were tried to obtain properly substituted cyclopentanyl aldehyde **90** in a large scale. Employing olefination with sulfonyl tetrazole **81**, as previously done, the unsaturated methyl ester **91** could be obtained in one step with a formidable *4R*-configured hydroxyl group. Conventional transformations, including deprotection and lactamization, finally afforded BFA lactam **92**.

**Scheme 20:**
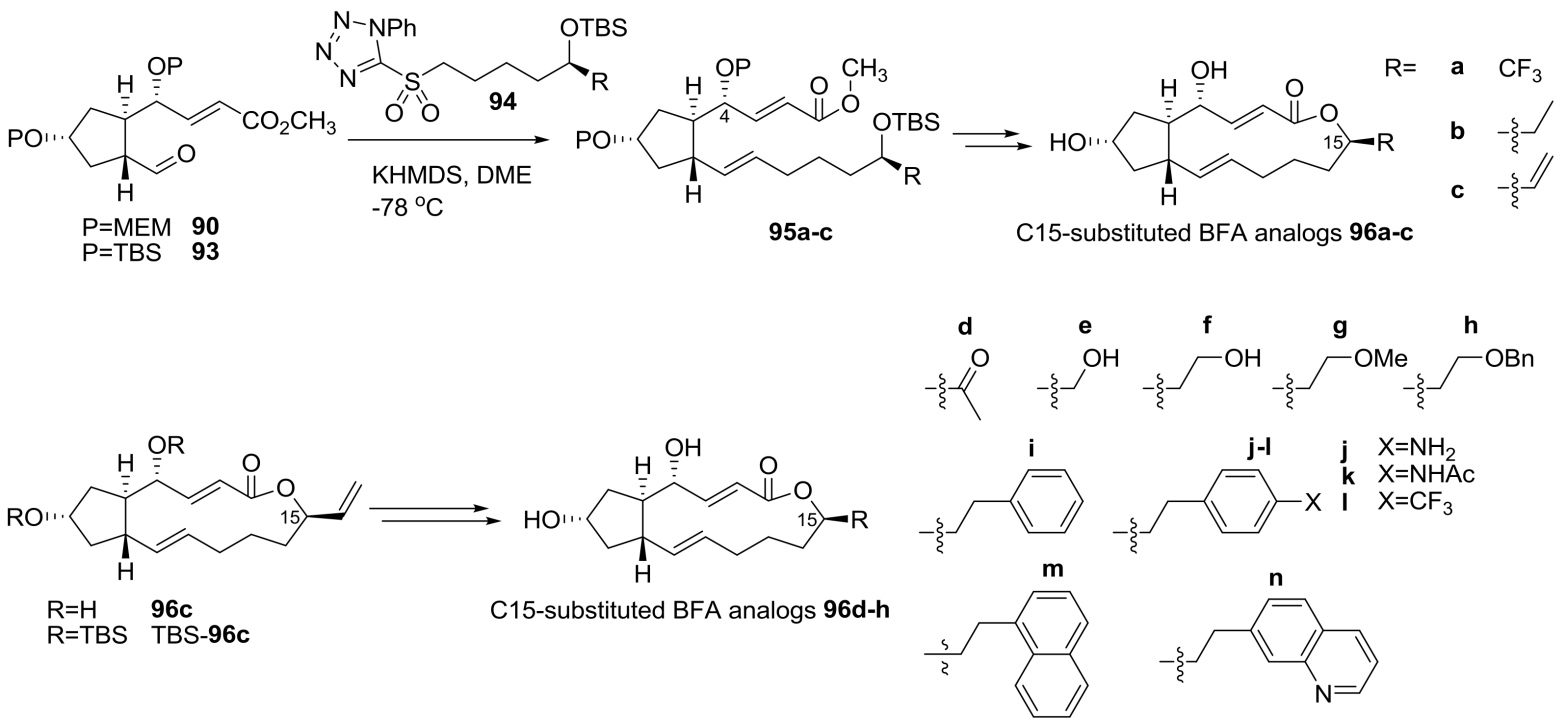
Synthesis of C15-substituted BFA analogs.

A similar semi-synthetic approach was also applied for the preparation of C15-substituted BFA analogs, as summarized in Scheme 20 [34]. The C15 position had been so far hardly changed because it did not have any active functional group. Employing this semi-synthesis however, it was possible to efficiently introduce unprecedented modifications at the C15 methyl group of BFA. The preparation of variously substituted sulfonyl tetrazoles **94**, followed by Julia–Kocienski olefination with *bis*-protected aldehyde **90** or **93** from BFA, produced a versatile synthesis of substituted ester **95** in good yield. Finally, deprotection–esterification afforded the C15-substituted BFA **96a**–**c** analogs uneventfully. The vinyl group at C15 in analog **96c** was also utilized for further transformation. Wacker oxidation, Suzuki coupling, or hydroboration was applied to add a carbonyl, an aromatic, or a hydroxyl group, respectively. After various transformation stages, 15 analogs were prepared for SAR study. Some of them are represented below.

The biological evaluation of the synthesized analogs was performed using 60 cancer cell lines, such as lung, colon, CNS, prostate, renal, ovarian, and breast cells, as well as leukemia and melanoma cells from the National Cancer Institute (Table 4). While some analogs did not show impressive anticancer activity, **96b**, **c**, **i**, and **j** showed potent cell growth inhibition. Importantly, these analogs showed more potent anticancer activity in specific cancer cells such as breast cancer cells. It means that advances in the modification of BFA based on medicinal chemistry and SAR study are continuing successfully.

**Scheme 21:**
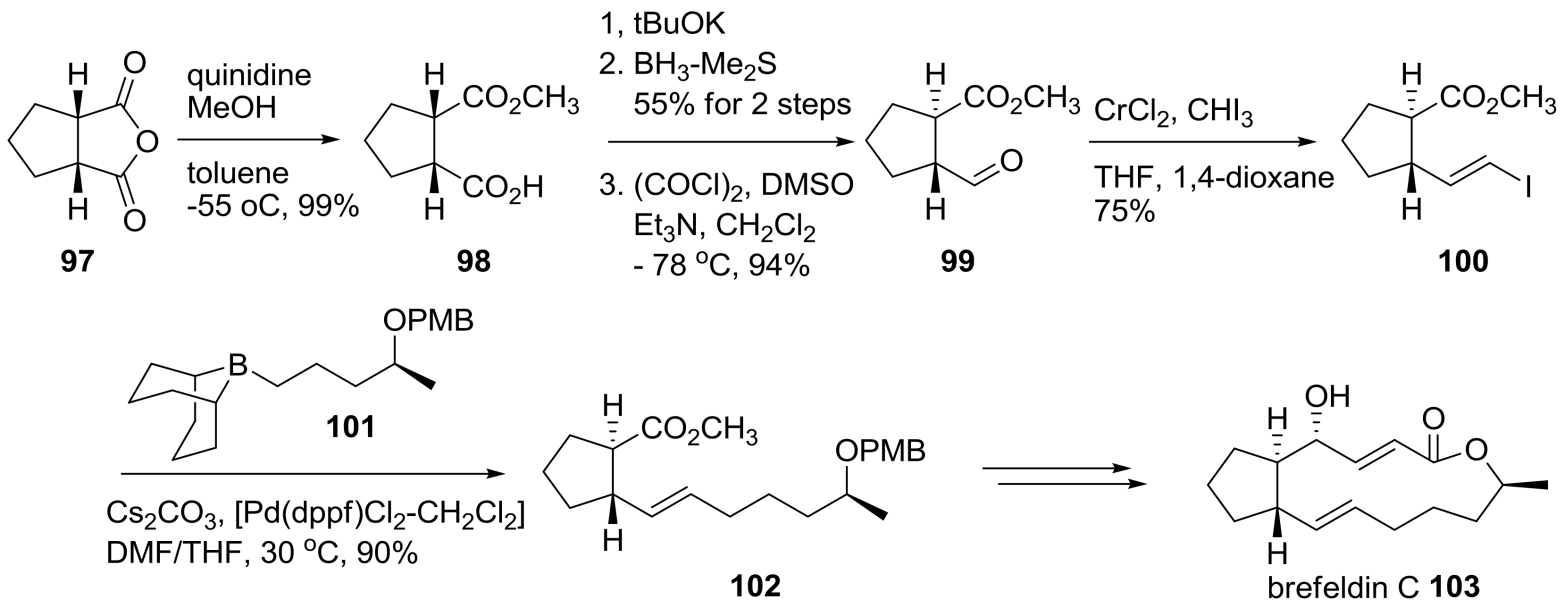
Synthesis of brefeldin C and its analogs. PMB: para-methoxybenzyl; dppf: 1,1′-*bis*(diphenylphosphino)ferrocene.

An asymmetric desymmetrization route of the BFA skeleton was also developed, as summarized in Scheme 21 [35]. Cyclopentylsuccinic anhydride **97** was submitted to asymmetric methanolysis using quinidine, a chiral natural amine, for the stereogenic differentiation of two carbonyl groups. A resulting chiral carboxylic acid was treated for epimerization, reduction, and Swern oxidation to produce *trans*-substituted cyclopentane **99** in good yield. After Takai olefination of aldehyde **99**, followed by Suzuki coupling with chiral borane **101**, *trans*-alkene **102** was obtained. By employing three carbon homologations, using acetylide addition or NHK reaction and usual transformations, brefeldin C **103** could be obtained successfully.

**Scheme 22:**
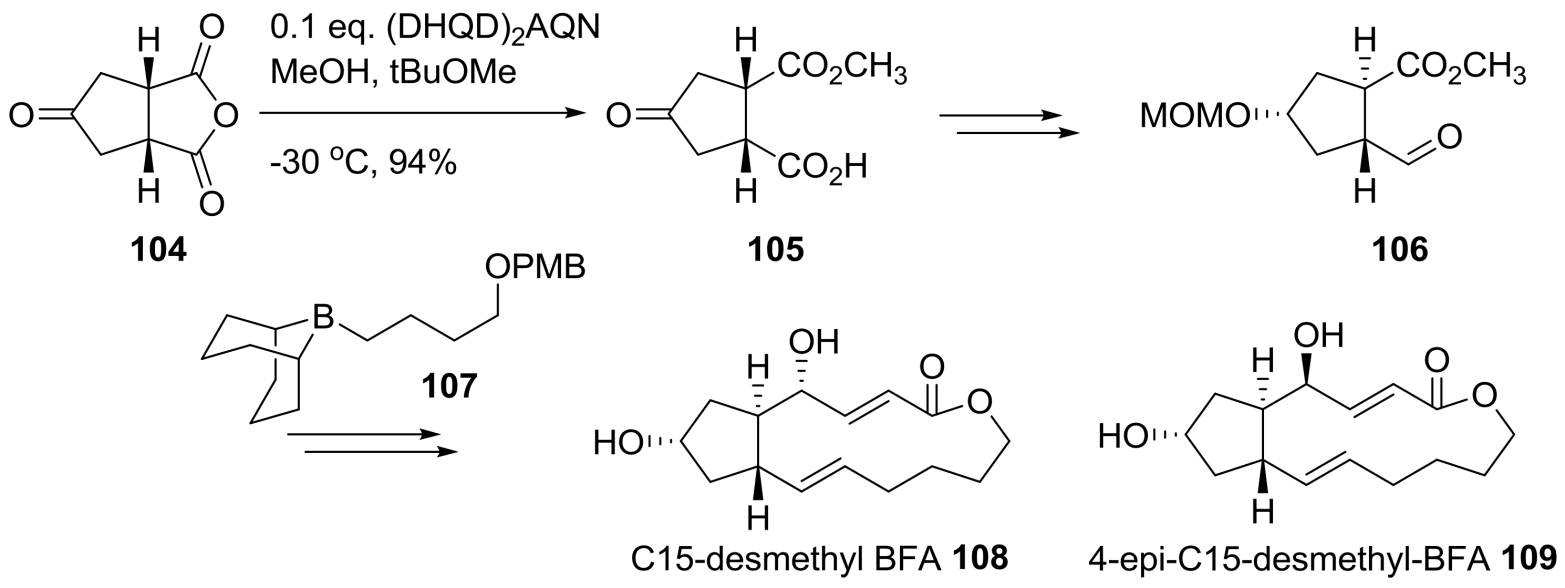
Synthesis of C15-desmethyl BFA analogs. (DHQD)_2_AQN: Hydroquinidine (anthraquinone-1,4-diyl) diether.

This strategy was also utilized to synthesize desmethyl BFA analogs **108** and **109** [36], as shown in Scheme 22. By employing the same strategy for chiral desymmetrization of anhydride **97**, oxocyclopentyl anhydride **104** could be efficiently converted to chiral ketone **105**. Instead of **101**, desmethyl side chain **107** was used to synthesize C15-desmethyl BFA analogs **108** and **109**, following the procedure developed in Scheme 21.

**Scheme 23:**
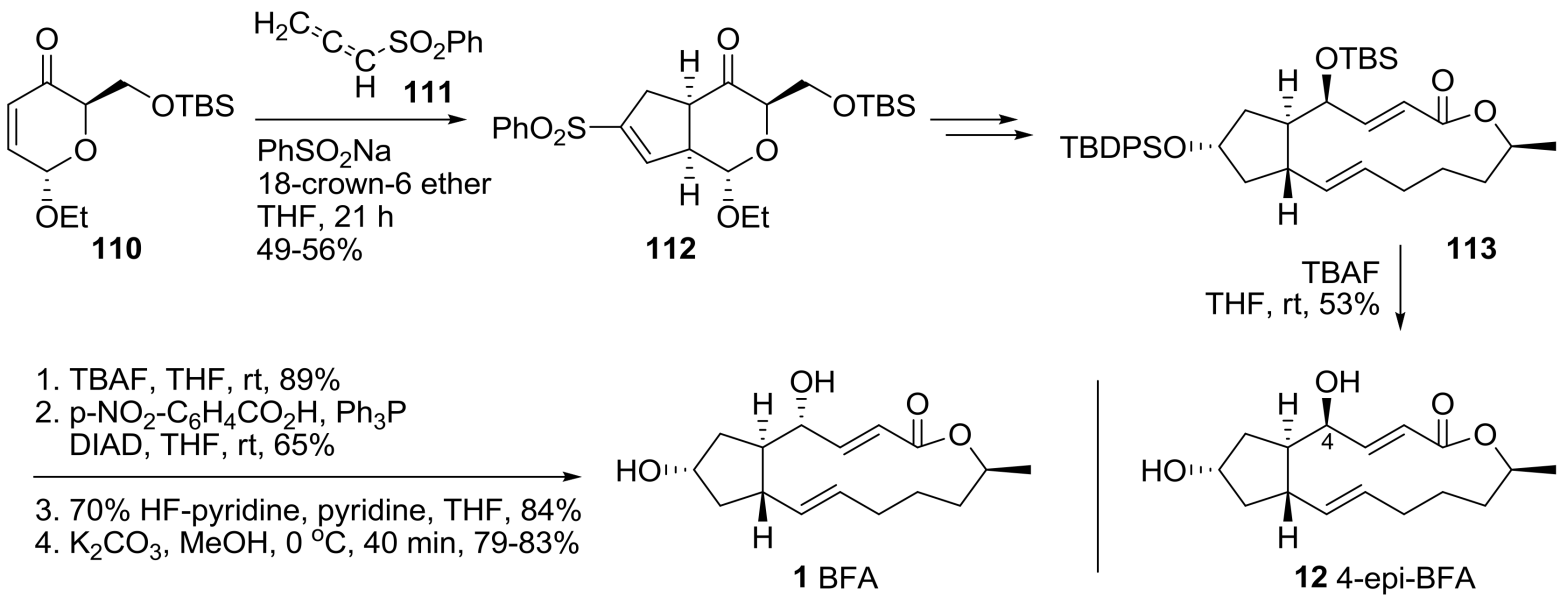
Synthesis of BFA and its 4-epimer through cycloaddition of allenylsulfone strategy.

A recent synthetic study employing a [3+2]-cycloaddition route was reported to enable a versatile synthesis of BFA **1** and 4-epi-BFA **12** from BFA, in a unified strategy [37]. In Scheme 23, cycloaddition of unsaturated enone **110** with allenylsulfone **111** afforded cyclopentane **112** in a stereoselective manner. After conversion of sulfonylalkene to the corresponding ketone, and following the reaction route, *bis*-protected BFA skeleton **113** could be obtained efficiently. Finally, BFA **1** and its C4-epimer **12** were prepared in the reaction conditions shown below. Direct global deprotection of **113** afforded 4-epi-BFA **12** in moderate yield, while selective TBS deprotection, followed by Mitsunobu inversion and sequential deprotection, yielded BFA **1** in a straightforward manner. With this synthetic route developed, a more promising synthetic analog of BFA can be anticipated in the future.

## 3. Conclusions

The apoptotic activity of BFA has been deeply researched and studied from a medicinal chemistry point of view. Based on an early report that a simple esterification of BFA still maintains its biological activity, a lot of prodrugs or esterified analogs, such as Nitric oxide-releasing BFA and long linker-containing BFA have been synthesized. Although some analogs showed better activity than BFA, the problem related to its physical properties is not solved yet, despite numerous advances in this sense. Hopefully, more soluble sulfide-conjugated BFA analogs have been developed for the purpose of producing BFA prodrugs. In addition, advanced synthetic technologies have been developed to more efficiently obtain the BFA skeleton. Studies on the mechanism of action of BFA were also carried out using these BFA analogs. By employing molecular studies it was shown that esterified BFA analogs were tolerated when inserting between ADP ribosylation factor (ARF) and ARF nucleotide binding site opener (ARNO) proteins, although this molecular interaction did not correspond to the disruption of the Golgi complex [24]. Co-crystalization of synthetic analogs with their target proteins would give meaningful information on their mode of action [38,39]. This synthetic and mechanistic studies would make it possible to change the BFA skeleton efficiently and provide more promising analogs of BFA that possess enhanced apoptotic activity in cancer cells.

## Figures and Tables

**Table 1:** Biological activity of the BFA analogs **2**–**15**.

Compound	EC ^1^ (µM)	IC_50_^2^ (µM)	Compound	EC (µM)	IC_50_ (µM)
**1** BFA	0.11	0.2	**9**	ND	-
**2**	0.27	0.1	**10**	4.1	20
**3**	0.27	2.0	**11**	ND	-
**4**	0.16	0.2	**12**	70	60
**5**	ND ^3^	-	**13**	ND	-
**6**	ND	-	**14**	ND	-
**7**	ND	3.0	**15**	ND	-
**8**	ND	-			

^1^ The effective concentration (EC) was determined by identification of DNA ladder in HCT-116 cells; ^2^ Based on MIT assay; ^3^ Not determined.

**Table 2:** Biological activity of BFA and its analogs **16**–**33**.

Compound	MGM ^1^ of GI_50_ (µM)	Compound	MGM of GI_50_ (µM)
**16**	4.1 ± 0.076	**25**	0.35
**17**	3.2 ± 0	**26**	0.037 ± 0.007
**18**	0.68 ± 0.062	**27**	0.20 ± 0.07
**19**	20 ± 1.8	**28**	0.11 ± 0.07
**20**	2.5	**29**	0.055 ± 0.028
**21**	1.8 ± 0.18	**30**	0.096 ± 0.077
**22**	3.00 ± 0.31	**31**	0.84 ± 0.61
**23**	36.3	**32**	1.47 ± 0.88
**24**	38	**33**	0.028 ± 0.017
**1** BFA	0.040 ± 0.019	**34**	0.048 ± 0.023

^1^ MGM: mean graph midpoint values of HOP-62 (lung), HCT-116 (colon), SF-539 (CNS), UACC-62 (melanoma), OVCAR-3 (ovarian), SN12C (renal), DU-145 (prostate), and MDA-MC-435 (breast) cell lines.

**Table 3:** Biological activity of BFA and its ester analogs.

Compound	MGM ^1^ of GI_50_ (µM)	Compound	MGM of GI_50_ (µM)
**1** BFA	0.04 ± 0.019	**45**	0.47 ± 0.001
**42**	13.00	**46**	5.10
**43**	97.72	**50**	29.51
**44**	0.11 ± 0.003	**51**	0.16 ± 0.002

^1^ MGM: mean graph midpoint values of A-549 (lung), HCT-116 (colon), SF-539 (CNS), UACC-62 (melanoma), OVCAR-3 (ovarian), SN12C (renal), PC3 (prostate), and MCF7 (breast) cell lines.

**Table 4:** Biological activity of the BFA and its ester analogs.

Compound	GI_50_ (µM) ^1^	TGI (µM) ^2^	Compound	GI_50_ (µM)	TGI (µM)
**1** BFA	0.021	3.48	**96h**	0.081	5.05
**96a**	0.321	5.49	**96i**	0.030	2.04
**96b**	0.045	5.82	**96j**	0.034	2.68
**96c**	0.032	4.91	**96k**	0.152	5.70
**96d**	0.593	13.35	**96l**	0.21	5.97
**96e**	2.570	38.00	**96m**	0.074	2.28
**96f**	0.178	7.69	**96n**	0.261	7.61
**96g**	0.090	5.78			

^1^ 60 cancer cell lines from the National Cancer Institute were tested. GI_50_ (growth inhibition 50%) was evaluated in two different 5-dose tests, except for **96e** (one 5-dose test); ^2^ TGI: total growth inhibition, i.e., concentration leading to of 100% of cell growth inhibition.
